# Can high-quality palliative care for respiratory patients be improved?

**DOI:** 10.1186/2049-6958-7-19

**Published:** 2012-07-20

**Authors:** Nicolino Ambrosino

**Affiliations:** 1 Pulmonary Rehabilitation and Weaning Center, Auxilium Vitae, Volterra, Pisa, Italy; 2 Pulmonary Unit, University Hospital, Pisa, Italy

## 

Respiratory diseases, including chronic obstructive pulmonary disease (COPD), are major causes of mortality worldwide [1], therefore high-quality palliative care for these patients is an important priority and a relevant component in the comprehensive management of these patients. Unfortunately, there is evidence that compared with cancer, patients with end-stage respiratory diseases receive poor-quality palliative care [2]. Among the reasons there is a bad or late patient-physician communication about end-of-life care.

In this issue of the Journal Vitacca and Comini [3] analyze the characteristics of respiratory patients who die in a pulmonary rehabilitation Unit dedicated to advanced care, evaluating the organizational support related to the process of dying and quality of care in the last days and hours of life. Among other interesting results, the authors report that despite patient-doctor communication was deemed to be good in the majority of the cases, and patient’s and family wishes to improve their relation were obtained in an high percentage, only a minority of patients’ relatives reported to have had a discussion about end-of-life care with their physicians.

Understanding and improving patient-clinician communication about end of- life care is the key to high-quality palliative care [4]. Usually the quality of this communication is likely to be poor and current models for training students, physicians, nurses and non professional care-givers in communication about end-of-life care are inadequate. Therefore the need to improve such training is maybe even greater than the need for better technical skills. Improvement in communication among doctors, nurses, patients and family may contribute to a perception of better quality of care and help the patients to fit with disease and its management leading to better satisfaction and even clinical outcomes. Communication skills for appropriate communication and facing emotional challenges may vary among persons and within the same person according to different levels of cognitive and emotional competence but can be learned. Understanding the barriers to this communication may be an important step to improving.

Anxiety and depression may be important obstacles for frank and clear discussion on palliative and end-of-life care. In the frame of end-of-life care programs treatment of depression with drugs and/or psychological education, behavioural therapy, group and individual counselling may be useful.

According to ethical and deontological principles in the frame of a medicine driven by agreed choices, doctors should respect patient’s willingness on the basis of either an informed consent or advanced directives. Advance directives and good advance care planning may represent an opportunity for improving the quality of palliative care for these patients. Nevertheless this mark of civilization is not available in all countries and somewhere patients are left at the mercy of doctors concerned more not to shorten life than to improve quality of life/death.

Hospice and palliative care services represent an important opportunity for improving end-of-life care. Such services are again not available in all countries, and may be differently located within the same country. Furthermore clinicians caring for patients with chronic pulmonary disease not always know the best ways to utilize these services.

In conclusion clinical decisions on end-of-life care must take into consideration the patient’s clinical and physical circumstances, preferences and likely actions to establish what treatment choice is available. Finally, clinical expertise makes the synthesis of all these considerations and recommends the treatment that the patient prefers [5]. Therefore the patient’s perception of quality of care is crucial and may be deeply influenced by media [6]. The paper by Vitacca and Comini helps us in this task.
